# Evidence for localised HIV related micro–epidemics associated with the decentralised provision of antiretroviral treatment in rural South Africa: a spatio–temporal analysis of changing mortality patterns (2007–2010)

**DOI:** 10.7189/jogh.04.010403

**Published:** 2014-06

**Authors:** Paul Mee, Mark A. Collinson, Sangeetha Madhavan, Elisabeth Dowling Root, Stephen M. Tollman, Peter Byass, Kathleen Kahn

**Affiliations:** 1 Medical Research Council/Wits University Rural Public Health and Health Transitions Research Unit (Agincourt), School of Public Health, Faculty of Health Sciences, University of the Witwatersrand, Johannesburg, South Africa; 2 WHO Collaborating Centre for Verbal Autopsy, Umeå Centre for Global Health Research, Umeå University, 90187 Umeå, Sweden; 3 Umeå Centre for Global Health Research, Division of Epidemiology and Global Health, Department of Public Health and Clinical Medicine, Umeå University, Umeå, Sweden; 4 INDEPTH Network, Kanda, Accra, Ghana; 5 Department of African–American Studies, University of Maryland, Maryland, USA; 6 Department of Geography and Institute of Behavioral Science, University of Colorado at Boulder, Boulder, Colorado, USA

## Abstract

**Background:**

In this study we analysed the spatial and temporal changes in patterns of mortality over a period when antiretroviral therapy (ART) was rolled out in a rural region of north–eastern South Africa. Previous studies have identified localised concentrated HIV related sub–epidemics and recommended that micro–level analyses be carried out in order to direct focused interventions.

**Methods:**

Data from an ongoing health and socio–demographic surveillance study was used in the analysis. The follow–up was divided into two periods, 2007–2008 and 2009–2010, representing the times immediately before and after the effects on mortality of the decentralised ART provision from a newly established local health centre would be expected to be evident. The study population at the start of the analysis was approximately 73 000 individuals. Data were aggregated by village and also using a 2 × 2 km grid. We identified villages, grid squares and regions in the site where mortality rates within each time period or rate ratios between the periods differed significantly from the overall trends. We used clustering techniques to identify cause–specific mortality hotspots.

**Findings:**

Comparing the two periods, there was a 30% decrease in age and gender standardised adult HIV–related and TB (HIV/TB) mortality with no change in mortality due to other causes. There was considerable spatial heterogeneity in the mortality patterns. Areas separated by 2 to 4 km with very different epidemic trajectories were identified. There was evidence that the impact of ART in reducing HIV/TB mortality was greatest in communities with higher mortality rates in the earlier period.

**Conclusions:**

This study shows the value of conducting high resolution spatial analyses in order to understand how local micro–epidemics contribute to changes seen over a wider area. Such analyses can support targeted interventions.

South Africa is one of the countries worst affected by the HIV pandemic. In 2011 there were estimated to be 5.6 million people infected with HIV, with an HIV prevalence of 17.6% for those aged between 15 and 49 [1]. In recent years the outlook for those infected has improved considerably with the percentage of those requiring antiretroviral treatment (ART) who are receiving the drugs increasing from 30% in 2009 [2] to 75.2% in 2011 [3]. The impact of ART programmes in reducing HIV related mortality and increasing life expectancy has been reported in a number of studies in sub–Saharan Africa [4–7]. However, the analysis of trends at a national, regional or district level may mask important local variations. It is important to understand this micro–level variation in order to effectively target interventions [8–12] as behaviours linked to higher or lower risk may be clustered in local communities [13,14].The data required for such detailed analyses are often only available in sites where the dynamics of a population can be followed in detail over time. Hence, Health and Demographic Surveillance Sites (HDSSs) provide the ideal platform for such analyses. [15,16]. Wand and Ramjee [10,11,14] identified local high prevalence spatial clusters of HIV and sexually transmitted infections amongst women in the Greater Durban area of Kwa Zulu Natal in South Africa. Tanser and colleagues [12] reviewed data from studies in various sub–Saharan African countries to provide evidence for the likely benefit of targeted interventions aimed at high risk groups to supplement more general population level approaches to HIV prevention.

Here we describe changing spatial patterns in the local development of an HIV epidemic over a period of time during which a health centre was established in order to test and treat those infected with HIV in addition to providing general health care for the local community. The goal of this analysis was to gain an understanding of which communities and sub–communities have benefitted preferentially from the enhanced access to treatment and those for which the situation may have deteriorated. By use of a grid overlay we were able break down communities into smaller sub–units and calculate cause–specific mortality trends in each of the grid squares. We also used clustering techniques to obtain complementary information on areas and periods of time where mortality risks differed from those expected. Clustering methods have been used previously to investigate HIV related mortality patterns in a rural South African setting, indicating the value of the technique in identifying locations for targeted interventions [8,17]. Using these multiple methodologies we were able to gain a fine grained understanding of the localised epidemic dynamics.

## METHODS

### Location of the study

**Figure 1:**
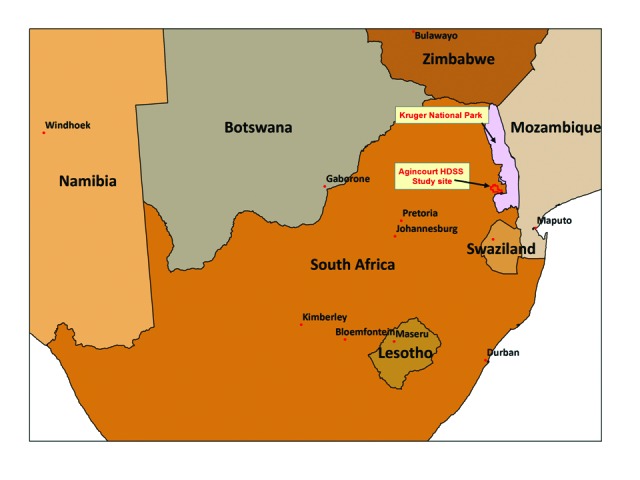
The location of the Agincourt HDSS in the South Africa and the surrounding region.

This study was carried out in the Agincourt Health and Socio–Demographic Survey site (AHDSS) located in the Bushbuckridge sub–district of Ehlanzeni municipality of Mpumalanga province in South Africa. [18] The site runs alongside the Kruger National Park and is close to the border with Mozambique (**Figure 1**). Approximately one third of the population are of Mozambican ethnic origin, mainly refugees from the Mozambican civil war. A number of the pre–existing villages gave over land in which ‘Mozambican’ settlements were established. These were initially characterised as having poorer levels of infrastructure and greater poverty than the established villages [19]. These former refugee communities are mainly located in the eastern part of the study site.

The area is predominantly rural though close to a number of peri–urban settlements. It is characterised as having high unemployment, a high level of poverty and relatively poor levels of educational attainment [18]. This has resulted in a high level of out–migration for economic reasons. Often the migrants retain strong connections with their original households and financial remittances from migrants are an importance source of income [20].

The baseline census for the AHDSS was carried out in 1992, and the present annual cycle of household follow–up visits was established in 1999. In 2007 the study site population was approximately 73 000 individuals. In order to maintain anonymity alphabetical identifiers rather than names are used to identify the villages in this study.

### Population distribution

**Figure 2:**
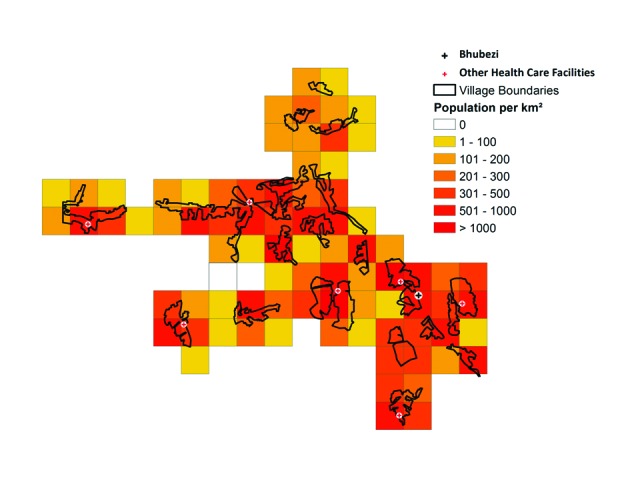
Mean population densities within the study site, 2007–2010.

The study site is made up of discretely bounded villages (**Figure 2**). Central areas of the villages often have a maximum population density greater than 1000 people/km^2^ which is more typical of an urban area in this region. The northern portion of the study site generally has a lower population density than the western and central parts.

### ART and VCT provision in the Agincourt site

Effective ART became available worldwide in the mid 1990s. In South Africa, however, the treatment only became available in the public sector in 2004 [2]. Subsequently there were delays in rolling out ART across the country particularly in rural areas such as that in which the Agincourt HDSS is located.

In 2002 a programme was introduced to provide voluntary counselling and HIV testing (VCT) services in the 5 health centres operating in the study area [21]. Prior to this VCT had only been available outside the site. Two of the secondary level hospitals serving the population in the study site began to provide ART treatment between 2004 and 2005. To improve clinic access a programme of decentralisation of ART services began in 2008 when a clinic in the study site (Agincourt) and another in a peri–urban settlement (Thulamahashe) just to the west of the site started to provide ART. At the same time a community health centre, Bhubezi, initially operating outside the public sector and providing general health care with an emphasis on HIV testing and treatment was established. Throughout this period ART was available from private providers.

This study examines changes in mortality in two consecutive time periods 2007–2008 and 2009–2010 the periods immediately before and after the possible effects on mortality of the decentralised ART provision from the Bhubezi health centre would be expected to be evident.

### Data used in the study

The core information captured in the annual AHDSS rounds were updates of the residency status and vital information for all household members. This was collected by interviewing the most knowledgeable available representative. For all deaths reported, a detailed verbal autopsy (VA) interview was carried out. The latitude and longitude of every dwelling was captured. Residents of two villages which were added to the site in 2009 were excluded from the analysis. The study population was restricted to males and females aged 15 years and above.

Cause of death (CoD) was assigned using the InterVA–4 model [22,23]. The InterVA–4 input variables are based on the questions in the standardised World Health Organisation (WHO) VA instrument released in 2012 [24]. A computer implementation of the InterVA–4 probabilistic model is freely available for download from http://www.interva.net . InterVA–4 uses a Bayesian probabilistic technique to calculate the likelihood of a particular cause of death for an individual based upon the presence or absence of particular signs or symptoms. An important advantage of the InterVA model over clinicians’ assessments is the consistency in cause of death ascertainment over time. A multicentre validation study of the InterVA–4 model against known HIV serostatus has shown good validity [25].

The variables required for the InterVA–4 input were derived from the responses given to the Agincourt VA questionnaire. In addition to directly matching variables, key words or phrases from narrative fields which were specifically associated with InterVA–4 input variables were identified. To overcome misspelling in the narratives, a string similarity matching algorithm (Jaro–Winkler) [26] was applied to identify similar strings, a cut–off score of 0.9 was used to define a match. These matches were checked manually. The matching algorithms were programmed using routines implemented in T–SQL, the proprietary implementation of the SQL standard in the SQL*Server^TM^ 2008 software package. [Microsoft Corporation, Redmond, Washington USA].

Figures from 2009 [27] indicate that around 70% of those infected with tuberculosis are co–infected with HIV in South Africa. Due to this high level of co–morbidity deaths due to HIV–related disease (WHO VA code –01.03 HIV/AIDS related death) and pulmonary tuberculosis (WHO VA code –01.09) were joined in a single category, HIV/TB. All other deaths for which a CoD was available were categorised as non–HIV/TB.

### Analytical approach

Mortality rates were directly standardised by age and gender in each 2-year period using the age/gender proportions of the entire site in the relevant period as the standard. The adult population was subdivided into 3 categories for the standardisation; 15 to 49, 50 to 65 and over 65. The standardised rates and the associated 90% confidence intervals (CI) were calculated using Stata, version 10.0 SE (Stata Corp., College Station, Texas, USA). Chi–Squared values and Student T–tests were used to compare the effect of indeterminate cause of death data.

An initial comparison of mortality patterns aggregated the individuals into their villages of residence. This approach was based on that of previous studies [28,29] where mortality clusters were identified using village centroids. A rationale for this approach is that within the study site each village is geographically isolated from the others with its own leadership which to some extent can influence local characteristics. However there is also likely to be intra–village heterogeneity which such an analysis may not identify. A study of national HIV prevalence data in South Africa [9] emphasised the danger in aggregating data only by province as this led to a loss of understanding of localised prevalence levels for communities which crossed provincial boundaries. A similar problem exists with village–level aggregation. For this reason, a grid was created extending across the site. After investigating various grid sizes a 2 × 2 km grid was chosen in order to give reasonable numbers of deaths and strata–specific populations in each square. A previous study of mortality patterns in the Butajira HDSS site in Ethiopia used a similar approach in order to get a more fine grained understanding of spatio–temporal mortality patterns [30].

The person years at risk were calculated for each individual in each of the two year time periods using the start of the period to begin the residence episode and either an end event, out–migration, death or the end of the period to right–censor the person time accrued. Mortality rate ratios were calculated for each grid square or village. In the grid analysis, if the lower bound of the 90% CI of the rate or rate ratio was higher than the mean value for the site that was classified as a significantly high value. Similarly if the upper bound was less than the mean this was classified as a grid square with a significantly low value. The grid and population density maps were developed using the ArcGIS software (ESRI 2011. ArcGIS Desktop: Release 10. Redlands, CA: Environmental Systems Research Institute). Other studies have suggested that lengthy travel times to clinics can provide a significant barrier to access [31–33], conversely better road links may also lead to greater opportunities for social mixing and hence a higher risk of exposure to infection [8]. Hence we assessed the proximity of areas of high or low mortality rates or rate ratios to the clinics and roads.

Spatio–temporal cluster analysis was carried out using Kulldorff’s spatio–temporal scan statistic as implemented in the SaTScan^TM^ software v9.1 to identify the location and evaluate the statistical significance of spatial and temporal clusters of mortality [34]. In this analysis a cylindrical scanning window is moved across the study area, so that each location of the window captures a unique set of individuals during a specific range of dates. The radius of each cylinder is allowed to vary. The height of the cylinder corresponds to a specific range of dates. To evaluate statistical significance, a comparison is made between the number of cases (eg, cause specific deaths) within the scanning window and the number of cases outside of that window using a maximum likelihood ratio test statistic. The centre of the scanning window moves in geographical space and time allowing significant high or low clusters of deaths in space and/or time to be identified. The population at each dwelling was derived from the total number of person years rounded to the nearest integer value with a minimum value of 1. The dates of death were aggregated by month. The maximum spatial and temporal windows used were 50% of the population at risk and 50% of the entire period (2 years) respectively. A Poisson probability model with a maximum of 999 Monte–Carlo replications was used in the analysis. A cut–off *P* value of 0.2 was used to identify clusters of interest. 

In order to obtain an objective measure of the degree of spatial homogeneity in the pattern of rate ratios, a calculation was made of the Global Moran’s I statistic using the GeoDa software [35]. A distance weight matrix was created using rook contiguity to indicate the nearest neighbours associated with a particular grid square. A Global Moran’s I statistic of close to zero indicates a random spatial distribution, values approaching +1 or –1 indicate that there is overall a high degree of spatial correlation.

## FINDINGS

Between 2007 and 2010, 3660 deaths were recorded in the study site. Of these, 567 (15.5%) were excluded from the analysis as a cause of death could not be assigned due to either the initial verbal autopsy not having been carried out or there being a lack of symptomatic data. For the remaining deaths, 2584 occurred in those aged 15 years and over. A further 13 deaths were excluded from the grid analysis as no geographical coordinates for the place residence of these individuals were available.

To assess the possible effects of indeterminate causes of death, an analysis was carried out of the spatial distribution of deaths with and without a CoD assignment for the entire study population. The spatial separation of the centroids of the home residences for the two categories was 464 m indicating that there was no significant spatial bias introduced. The percentage of indeterminate CoD assignments was greater in 2009 to 2010 than in 2007 to 2008 (16.3% compared with 15.7%, *P* = 0.014). There were more indeterminate CoDs for males than females (17.2% compared with 14.2%, *P* = 0.012). Also those with indeterminate CoDs were on average younger than those for whom a cause of death was assigned (36.7 years compared to 42.5 years, *P* < 0.001)

### Village level analysis of mortality

**Figure 3:**
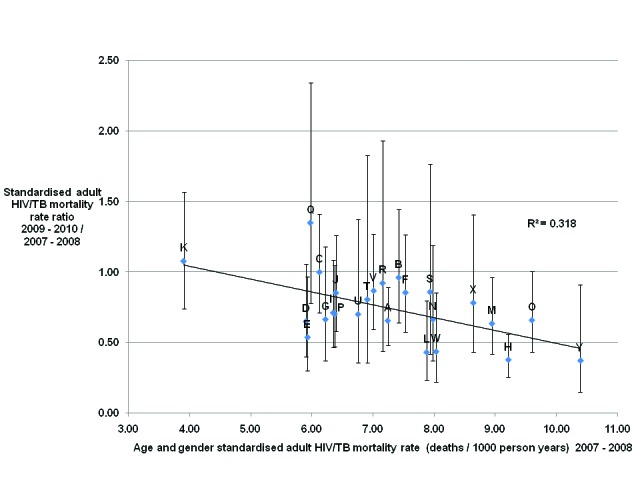
A comparison of the HIV/TB mortality rate ratios (with 90% confidence intervals) and mortality rates for 2007–2008 (points are labelled with the village codes). The best fit trend line is superimposed on the points with the associated R–squared value.

**HIV/TB mortality.** There was a large range of values for the standardised mortality rates (SMRs) for HIV/TB during both time periods (see Table S1 in the Online Supplementary Document(Online Supplementary Document)). The rate ratio between the latter and earlier period was 0.70 (90% CI = 0.64–0.77) indicating a decrease in the HIV/TB mortality rates of between 23% and 36% with a point estimate of 30%. The rate ratios (RR) for individual villages are shown in **Figure 3**.

A rate ratio greater than 1 indicating an increase in the adult HIV/TB mortality rates was seen for two of the twenty–five villages (Q and K.) For twenty–two villages a decrease in the HIV/TB mortality rates were seen. However, for fourteen of these the upper bound of the 90% CI was greater than or equal to 1 indicating that the evidence for a decrease was weak. For one village the HIV/TB mortality rate remained unchanged.

Village Q had the highest rate ratio value of 1.35 (90% CI = 0.78–2.34) indicating a point estimate of a 35% increase in the HIV/TB mortality rate, as the lower bound of the confidence interval is less than 1 it is possible that there was a trend towards a decrease in the rate over the period. The lowest HIV/TB mortality rate ratios were 0.37 (90% CI = 0.15–0.91) for village Y, 0.38 (90% CI = 0.26–0.56) for village H and 0.43 (90% CI = 0.23–0.80) for village L, in each case the upper bound of the confidence interval is less than 1 indicating a rate decrease to be a reasonable interpretation. Villages H and L are geographically close neighbours to village Q.

The gradient of the trend line fitted to the plotted points (**Figure 3**) was 0.091, indicating a decrease of close to 9% in the mortality rate ratio between the two periods for each unit increase in the mortality rate in the earlier period. The low R^2^ value of 0.318 showed that there was a significant amount of variation around this overall trend.

**Non–HIV/TB mortality.** Similar to HIV/TB deaths, there were large variations between villages in the SMRs for deaths due to causes other than HIV/TB during both time periods (see Table S2 in the Online Supplementary Document(Online Supplementary Document)). The standardised adult rate ratio for non–HIV/TB mortality between 2007 to 2008 and 2009 to 2010 was 1.01 (90% CI = 0.92–1.10) indicating that there was no overall change evident in the non HIV/TB mortality rates for adults between the two periods. The range in rate ratios was from 0.42 (90% CI = 0.25–0.71) for village N to 1.84 (90% CI = 1.19–2.86) for village W. With the upper bound of the rate ratio for village N less than 1 and the lower bound for village W greater than 1 it is reasonable to interpret this as evidence for significantly different changes in cause–specific rates between the two.

**Figure 4:**
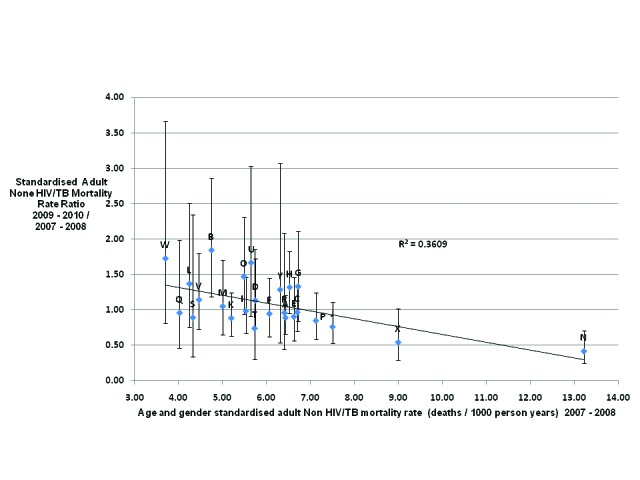
A comparison of the Non HIV/TB mortality rate ratios (with 90% confidence intervals) and mortality rates for 2007–2008 (points are labelled with the village codes). The best fit trend line is superimposed on the points with the associated R–squared value.

The graph in **Figure 4** shows a decrease in the rate ratio as the initial mortality rate increases with a gradient of 0.110 for the trend line. The R^2^ value of 0.361 again indicates significant variation around the overall trend.

### Gridded mortality plots

**Figure 5:**
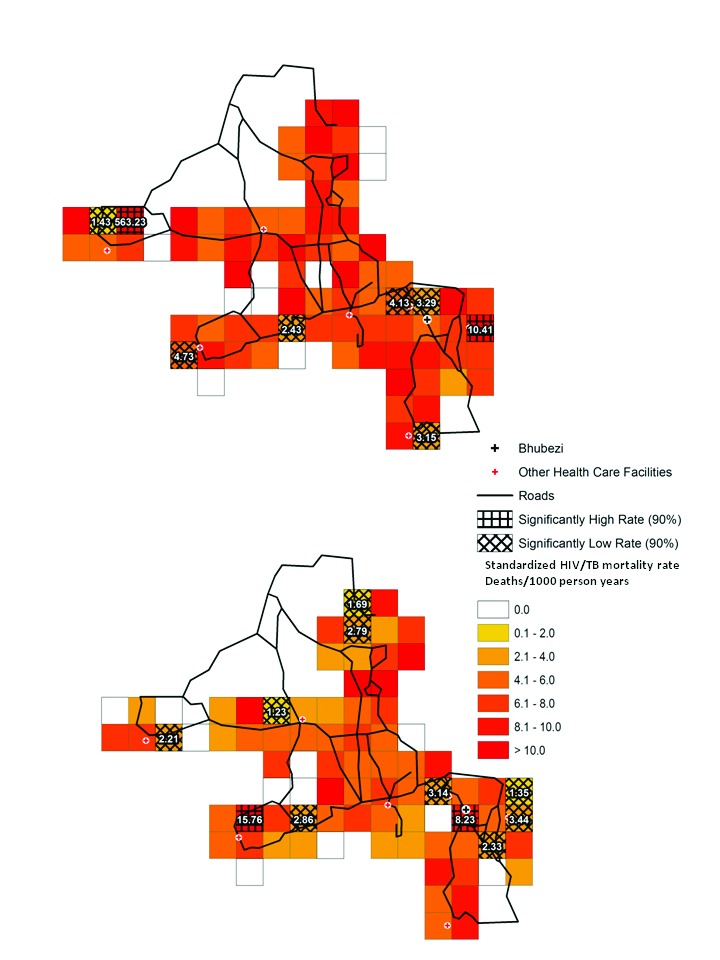
HIV/TB mortality rates (deaths per 1000 person-year, PY) from 2007 to 2008 (upper panel) and 2009–2010 (lower panel) in 2 km grid squares for the Agincourt HDSS site. For grid squares with significantly low rates, the upper bound of the 90% confidence interval is less than the mean rate. For grid squares with significantly high rates the lower bound of the 90% confidence interval is greater than the mean rate. The label is the mortality rate for the grid square.

**HIV/TB mortality.** **Figure 5** shows the HIV/TB mortality rates in each grid square for 2007 to 2008 and 2009 to 2010. In 2007 to 2008 (**Figure 5**, upper panel), only two grid squares with significantly high rates were seen one at the extreme east of the site (10.41 deaths/1000 person-years, PY), the other towards the west (563.23 deaths/1000 PY). The latter was in a square with a very low population and thus likely to be a statistical outlier. Grid squares with significantly low standardised adult HIV/TB mortality rates were predominantly in the southern and western areas of the site with two additional squares located to the north of the Bhubezi health community health centre (Bhubezi).

In 2009 to 2010 (**Figure 5**, lower panel) there were 4 grid squares with significantly low rates in the eastern region of the site in the area around Bhubezi. Other low rate grid squares were identified towards the west and north of the site. There were two grid squares with significantly high HIV/TB mortality rates one containing Bhubezi (8.23 deaths/1000 PY) and the other towards the southwest of the site (15.76 deaths/1000 PY)

**Figure 6:**
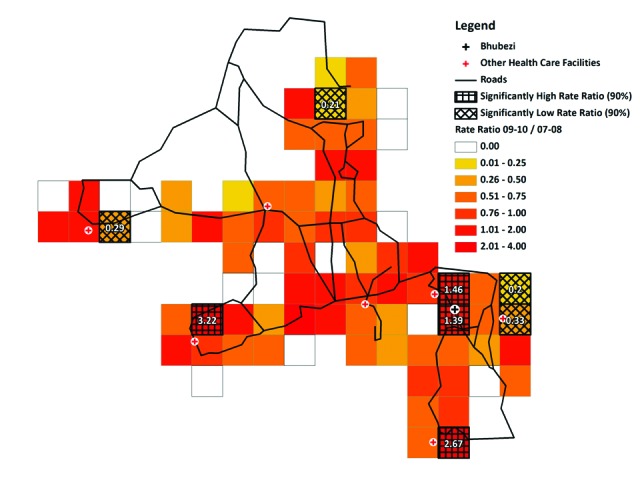
HIV/TB mortality rate ratios for 2009–2010 compared to 2007–2008 for the Agincourt HDSS site. For grid squares with significantly low rate ratios, the upper bound of the 90% confidence interval is less than the mean rate ratio. For grid squares with significantly high ratios, the lower bound of the 90% confidence interval is greater than the mean rate ratio. The label is the mortality rate ratio for the grid square.

The rate ratio plot (**Figure 6**) shows four grid squares with significantly high rate ratios indicating an increase in the adult HIV/TB mortality rates between the two periods. One was located in the square containing Bhubezi (rate ratio = 1.39) and another directly to the north (rate ratio = 1.46), another was in the extreme south of the site (rate ratio = 2.67) and one towards the west (rate ratio = 3.22). Of the four grid squares with significantly low rate ratios, two were located to the east of Bhubezi. The rate ratios for these two squares are 0.20 and 0.33, indicating decreases of 80% and 67% respectively in the adult HIV/TB mortality rates in these regions. Other grid squares with low mortality rate ratios were seen in the north and west of the site.

The Moran’s I value for the HIV/TB mortality rate ratios was –0.03 indicating the spatial distribution of changes in HIV/TB mortality rates was essentially random with no evidence for global spatial correlation.

**Non–HIV/TB mortality.** For adult deaths other than those caused by HIV related disease or TB, in the period 2007 to 2008, two grid squares with significantly high mortality rates were identified towards the north of the site and one in the south (see Figure S1 in Online Supplementary Document(Online Supplementary Document)). In contrast, low rate grid squares were mainly found in the eastern, western and central areas of the site. In 2009 to 2010 the four grid squares showing significantly high rates were located in the eastern, southern and central regions of the site. There were eight grid squares with significantly low rates. These were distributed throughout the site other than in the extreme southern and western areas.

Two grid squares had significantly high rate ratio values, one just below Bhubezi (rate ratio = 2.23) and one towards the centre of the site (rate ratio = 1.92). Five grid squares showed significantly low rate ratios, one was just to the north–east of Bhubezi the others towards the centre of the site.

The Moran’s I value for the non–HIV/TB rate ratios was –0.02 indicating the spatial variation in non–HIV/TB mortality rate changes was essentially random again showing no evidence for global spatial correlation.

### Spatio–temporal clustering

**Figure 7:**
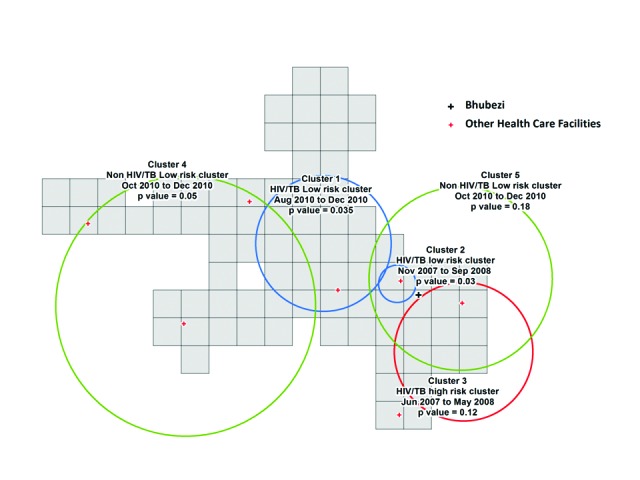
Spatio–temporal clusters with significantly raised or lowered rates of deaths due to HIV/TB and Non HIV/TB over the period 1 Jan 2007 to 31 Dec 2010.

For HIV/TB mortality two low risk clusters (1 & 2) and 1 high risk cluster (3) were identified as shown in **Figure 7**. Cluster 1 (*P* = 0.04) was located in the centre of the site the time period was from August to December of 2010. Cluster 2 (*P* = 0.03) to the southeast of Cluster 1 was between November 2007 and September 2008. Cluster 3 had a lower level of statistical significance (*P* = 0.12), its time period from June 2007 to May 2008 partially overlapped that for cluster 2. It was located in the lower southeast region of the site.

For the deaths due to causes other than HIV/TB, two low risk clusters were identified (4 & 5) over the same time period, October 2010 to December 2010. Cluster 4 (*P* = 0.05) covers a large area of the west and central region of the site, whilst cluster 5 with a low level of statistical significance (*P* = 0.18) covers a slightly smaller area towards the east of the site.

## DISCUSSION

In this study three complementary techniques were used to investigate the changes in the geographical patterns of cause specific adult mortality over a period in which ART was rolled out and a community health centre opened in a rural South African community.

There was a 30% decrease in adult HIV/TB mortality between 2007 to 2008 and 2009 to 2010. However, for one community, village Q, there was a 35% increase in the adult HIV/TB mortality rate over the same period. As the rates were age and gender standardised, other factors must explain these differences. Village Q was originally established to provide homes for Mozambican refugees, this suggested that the ethnic profile of the village might be an important factor in determining the take–up of ART and subsequent decrease in mortality. However in our study we also found that in other predominantly Mozambican communities, such as villages L, R, S and T, there were decreases in the HIV/TB mortality rate over the same period. Hence ethnicity alone cannot explain the mortality changes. Furthermore, village L neighbours village Q, highlighting the geographical heterogeneity in the epidemic trajectories seen for closely neighbouring communities. This heterogeneity in rates between villages is consistent with patterns in all cause mortality identified between 1992 and 2007 [36] in this area.

The inverse relationship between the adult mortality rate ratio and the baseline adult HIV/TB mortality rate suggests that at a population level the impact of ART was greatest in the communities where the need was greatest. It is possible the higher HIV/TB mortality rates in some communities increased the awareness of the disease and hence the likelihood of individuals in those areas getting tested and starting treatment. Further qualitative studies would be needed to confirm whether this was the case.

There was no evidence of overall changes in adult mortality rates due to causes other than HIV related disease and pulmonary TB between the two time periods. This suggests that the reduction in HIV/TB mortality was explained by the provision of ART, rather than a general improvement in other aspects of health care provision in the area. However we do see highly heterogeneous patterns of change between different communities. Also the patterns of change in mortality rates differ markedly for the two categories of cause of death. An example of this is seen for village O which had a 37% increase in non–HIV/TB mortality and a 34% decrease in HIV/TB mortality, between

The analysis by grid square further emphasises the pattern of local heterogeneity and shows how this can also be seen at a sub–village level. As an example, the four populated grid squares located towards the extreme west of the site in the map of adult HIV/TB mortality (**Figure 6**) are subsections of the same village. Three have increased HIV/TB mortality rates whilst the fourth shows a reduction of 71% between the earlier and later time periods. It is possible that the characteristics of the different sub–regions of the village are influenced by those of neighbouring communities.

The existence of clusters of high and low HIV/TB mortality towards the east of the study site (**Figure 7**) also shows the local heterogeneity in the risk of death related to these conditions. In comparison, a study looking at infant HIV/TB deaths in the site between 2000 and 2005 identified high mortality risk hot spots in the central, south–eastern and south–western areas [37]. This analysis shows different geographical locations for the high and low risk clusters of mortality attributable to different causes consistent with a previous study of adult mortality patterns in the site from 1993–2010 [38].

Visual analysis showed no evidence for lower HIV/TB rate ratios in areas close to the clinics providing ART. A study in a rural area of KwaZulu–Natal in South Africa showed that ART uptake was inversely related to the distance individuals lived from the clinic providing treatment [39]. Previous studies in this site did identify associations between the mortality risk and the straight-line distance between an individual’s residence and local clinics [40,41].

These patterns have important implications for those planning new health facilities or initiating community–based health interventions. Whilst it is important that the physical barriers to access are reduced, for example by locating new health centres in order to obtain the greatest reduction in travel times for the population which is served [31], consideration should also be made to the different morbidity patterns across the community. Furthermore as we see from the patterns of HIV/TB rate ratios in **Figure 6** different areas within a short distance of the Bhubezi health centre saw very different epidemic trajectories over the period of the study. This suggests that to have an effective impact, the various barriers to access experienced by different sub–sections of the community, must be addressed [42]. Clearly there are issues of temporality in such an approach as we are making assumptions about levels of current illness based on past mortality levels. Also knowing the place of residence of an individual at the time of their death does not necessarily tell you where the person was exposed to the risk factors leading to death. This is especially important in an area like this where we see significant numbers of individuals returning from urban areas when their illness has progressed such that they are no longer able to maintain employment and live independently [43].

A potential limitation in this analysis is the rather high level of indeterminate causes of death. However there is no evidence that the indeterminate data introduced a spatial bias. The preponderance of indeterminate causes among younger individuals and males probably reflects patterns of temporary migration, with a number of those deaths occurring away from home and hence difficult to follow–up by verbal autopsy. As there was a slightly higher proportion of deaths for which no cause could be defined in 2009–2010 compared to 2007–2008 (16.3% vs 15.7%) we can assume that the cause specific mortality ratios are a slight underestimate. Also as relatively short time windows were used to accumulate the data, the numbers of deaths accrued was relatively low compared to previous studies [36] leading to relatively wide confidence intervals for the rates and rate ratios calculated.

## CONCLUSIONS

The patterns that emerge from this study are complex. In some areas there appears to be a high level of intra–village coherence with individual villages showing distinct mortality characteristics which differ from those of neighbouring villages. In others the pattern is less clear. This work confirms the conclusions made by other studies in the region that identified heterogeneous pattern of micro–epidemics within a more generalised epidemic [10–12,14,17]. Our results confirm trends shown by Tanser and colleagues [8] of considerable local geographic variation in HIV prevalence in a rural area of Kwa Zulu Natal South Africa. However spatial patterns of HIV related mortality will be affected by both the distributions of risk factors for infection and local availability of testing and treatment. In our case there are no obvious associations with the location of major roads crossing the site. This study shows that a micro–level analysis may be useful in mounting an appropriate public health response to HIV in a local area. In a further study, currently being prepared for publication, we assess the influence of various risk factors which may explain the spatial patterns that have been identified. Conclusions drawn from a spatial analysis such as is presented here can be used as a starting point for investigations of factors influencing the differing current morbidity patterns seen in different communities.

Geo–located cause specific mortality data as used in this analysis is often lacking in resource poor regions [44]. However a mobile phone based application which can be used to collect verbal autopsy data and assign causes of death, as well as automatically registering GPS coordinates, is now available [45]. This gives the potential in the future for a greater geographic coverage of mortality data beyond the existing limited number of research sites for which it is available.

UNAIDS and others have emphasised that in deciding how to respond to HIV one must “Know your epidemic” [46]. This study emphasises the importance of that approach.

## References

[1] UNAIDS. UNAIDS report on the global AIDS epidemic 2012. United Nations, 2012. Available at: http://www.unaids.org/en/resources/publications/2012/name,76121,en.asp. Accessed: 10 May 2014.

[2] Ojikutu B, Makadzange AT, Gaolathe T (2008). Scaling up ART treatment capacity: lessons learned from South Africa, Zimbabwe, and Botswana.. Curr HIV/AIDS Rep.

[3] South African National AIDS Council, National Department of Health. Global AIDS response progress report 2012 Republic of South Africa. Available at: http://www.unaids.org/en/dataanalysis/knowyourresponse/countryprogressreports/2012countries/ce_ZA_Narrative_Report.pdf. Accessed: 13 May 2014.

[4] Herbst AJ, Cooke GS, Bärnighausen T (2009). KanyKany A, Tanser F, Newell M–L. Adult mortality and antiretroviral treatment roll–out in rural KwaZulu–Natal, South Africa.. Bull World Health Organ.

[5] Jahn A, Floyd S, Crampin AC, Mwaungulu F, Mvula H, Munthali F (2008). Population–level effect of HIV on adult mortality and early evidence of reversal after introduction of antiretroviral therapy in Malawi.. Lancet.

[6] Herbst AJ, Mafojane T, Newell M-L (2011). Verbal autopsy–based cause–specific mortality trends in rural KwaZulu–Natal, South Africa, 2000–2009.. Popul Health Metr.

[7] Bor J, Herbst AJ, Newell M-L, Bärnighausen T (2013). Increases in adult life expectancy in rural South Africa: valuing the scale–up of HIV treatment.. Science.

[8] Tanser F, Barnighausen T, Cooke GS, Newell ML (2009). Localized spatial clustering of HIV infections in a widely disseminated rural South African epidemic.. Int J Epidemiol.

[9] Kleinschmidt I, Pettifor A, Morris N, MacPhail C, Rees H (2007). Geographic distribution of human immunodeficiency virus in South Africa.. Am J Trop Med Hyg.

[10] Wand H, Whitaker C, Ramjee G (2011). Geoadditive models to assess spatial variation of HIV infections among women in Local communities of Durban, South Africa.. Int J Health Geogr.

[11] Wand H, Ramjee G (2010). Targeting the hotspots: investigating spatial and demographic variations in HIV infection in small communities in South Africa.. J Int AIDS Soc.

[12] Tanser F, de Oliveira T, Maheu–Giroux M, Barnighausen T (2014). Concentrated HIV subepidemics in generalized epidemic settings.. Curr Opin HIV AIDS..

[13] Aral SO, Padian NS, Holmes KK (2005). Advances in multilevel approaches to understanding the epidemiology and prevention of sexually transmitted infections and HIV: an overview.. J Infect Dis.

[14] Ramjee G, Wand H (2014). Geographical clustering of high risk sexual behaviors in “Hot–spots” for HIV and sexually transmitted infections in Kwazulu–Natal, South Africa.. AIDS Behav.

[15] Sankoh O, Byass P (2012). The INDEPTH Network: filling vital gaps in global epidemiology.. Int J Epidemiol.

[16] Kaufman JS, Asuzu M, Rotimi C, Johnson O, Owoaje E, Cooper R (1997). The absence of adult mortality data for sub–Saharan Africa: a practical solution.. Bull World Health Organ.

[17] Namosha E, Sartorius B, Tanser F (2013). Spatial Clustering of All–Cause and HIV–Related Mortality in a Rural South African Population (2000–2006).. PLoS ONE.

[18] Kahn K, Collinson M, Gómez–Olivé F, Kabudula CWMP, Mokoena O, Shabangu M (2012). Profile: Agincourt health and socio–demographic surveillance system.. Int J Epidemiol.

[19] Polzer Ngwato T (2012). Together apart: migration, integration and spatialised identities in South African border villages.. Geoforum.

[20] Collinson MA (2010). Striving against adversity: the dynamics of migration, health and poverty in rural South Africa.. Glob Health Action..

[21] Pronyk PM, Kim JC, Makhubele MB, Hargreaves JR, Mohlala R, Hausler HP (2002). Introduction of voluntary counselling and rapid testing for HIV in rural South Africa: from theory to practice.. AIDS Care.

[22] Byass P, Fottrell E, Dao LH, Berhane Y, Corrah T, Kahn K (2006). Refining a probabilistic model for interpreting verbal autopsy data.. Scand J Public Health.

[23] Byass P, Kahn K, Fottrell E, Collinson MA, Tollman SM (2010). Moving from data on deaths to public health policy in Agincourt, South Africa: approaches to analysing and understanding verbal autopsy findings.. PLoS Med.

[24] Byass P, Chandramohan D, Clark SJ, D'Ambruoso L, Fottrell E, Graham WJ (2012). Strengthening standardised interpretation of verbal autopsy data: the new InterVA–4 tool.. Glob Health Action..

[25] Byass P, Calvert C, Miiro–Nakiyingi J, Lutalo T, Michael D, Crampin A (2013). InterVA–4 as a public health tool for measuring HIV/AIDS mortality: a validation study from five African countries.. Glob Health Action..

[26] Winkler WE, editor. Overview of record linkage and current research directions. Bureau of the Census, 2006. Available at http//citeseerx.ist.psu.edu/viewdoc/summary?=10.1.1.79.1519. Accessed 10 May 2014.

[27] Abdool Karim SSA, Churchyard GJ, Karim QA, Lawn SD (2009). HIV infection and tuberculosis in South Africa: an urgent need to escalate the public health response.. Lancet.

[28] Sartorius B, Kahn K, Collinson MA, Vounatsou P, Tollman SM (2011). Survived infancy but still vulnerable: spatial–temporal trends and risk factors for child mortality in the Agincourt rural sub–district, South Africa, 1992–2007.. Geospat Health.

[29] Sartorius BK, Kahn K, Vounatsou P, Collinson M, Tollman S (2010). Young and vulnerable: Spatial–temporal trends and risk factors for infant mortality in rural South Africa (Agincourt), 1992–2007.. BMC Public Health.

[30] Byass P, Fantahun M, Emmelin A, Molla M, Berhane Y.  (2010). Spatio–temporal clustering of mortality in Butajira HDSS, Ethiopia, from 1987 to 2008.. Glob Health Action.

[31] Tanser F (2006). Methodology for optimising location of new primary health care facilities in rural communities: a case study in KwaZulu–Natal, South Africa.. J Epidemiol Community Health.

[32] Zachariah R, Harries AD, Manzi M, Gomani P, Teck R, Firmenich P (2006). Acceptance of anti–retroviral therapy among patients infected with HIV and tuberculosis in rural Malawi is low and associated with cost of transport.. PLoS ONE.

[33] Kunihira NR, Nuwaha F, Mayanja R, Peterson S (2010). Barriers to use of antiretroviral drugs in Rakai district of Uganda.. Afr Health Sci.

[34] Kulldorff M (1997). A spatial scan statistic.. Comm Stat Theory Methods.

[35] Anselin L, Syabri I, Kho Y (2006). GeoDa: an introduction to spatial data analysis.. Geogr Anal.

[36] Sartorius B, Kahn K, Vounatsou P, Collinson M, Tollman S. (2010). Space and time clustering of mortality in rural South Africa (Agincourt HDSS) 1992-2007.. Glob Health Action.

[37] Musenge E, Vounatsou P, Kahn K (2011). Space–time confounding adjusted determinants of child HIV/TB mortality for large zero–inflated data in rural South Africa.. Spat Spatiotemporal Epidemiol.

[38] Sartorius B, Kahn K, Collinson MA, Sartorius K, Tollman SM (2013). Dying in their prime: determinants and space–time risk of adult mortality in rural South Africa.. Geospat Health.

[39] Cooke GS, Tanser F, Bärnighausen T, Newell M-L (2010). Population uptake of antiretroviral treatment through primary care in rural South Africa.. BMC Public Health.

[40] Musenge E, Vounatsou P, Collinson M, Tollman S, Kahn K (2013). The contribution of spatial analysis to understanding HIV/TB mortality in children: a structural equation modelling approach.. Glob Health Action..

[41] Sartorius B (2013). Modelling determinants, impact, and space–time risk of age–specific mortality in rural South Africa: integrating methods to enhance policy relevance.. Glob Health Action..

[42] McIntyre D, Thiede M, Birch S (2009). Access as a policy–relevant concept in low–and middle–income countries.. Health Econ Policy Law.

[43] Clark SJ, Collinson MA, Kahn K, Drullinger K, Tollman SM (2007). Returning home to die: circular labour migration and mortality in South Africa.. Scand J Public Health Suppl.

[44] Byass P (2007). Who needs cause–of–death data?. PLoS Med.

[45] Bird J, Byass P, Kahn K, Mee P, Fottrell E, editors. A matter of life and death: practical and ethical constraints in the development of a mobile verbal autopsy tool; 2013. Proceedings of the SIGCHI Conference on Human Factors in Computing Systems. New York: ACM, 2013. P. 1489-98.

[46] Wilson D, Halperin DT (2008). “Know your epidemic, know your response”: a useful approach, if we get it right.. Lancet.

