# Supplementary material for: Evidence for localised HIV related micro–epidemics associated with the decentralised provision of antiretroviral treatment in rural South Africa: a spatio–temporal analysis of changing mortality patterns (2007–2010)
Source: J Glob Health. 2014 Jun;4(1):010403. doi: 10.7189/jogh.04.010403 (PMC4073250; doi:10.7189/jogh.04.010403)
Supplement: Online Supplementary Document [file jogh-04-010403-s001.pdf]

Online Supplementary Document
Mee P et al. Evidence for localised HIV related micro–epidemics associated with the decentralised provision of antiretroviral treatment in rural South Africa: a spatial–temporal analysis of changing mortality patterns (2007–2010)
Journal of Global Health 2014;4:010403

Table S1 Analysis of Age and Gender standardised AIDS/TB mortality rates and rate ratios aggregated by village for 2007-2008 and 2009-2010

	2007 - 2008				2009 – 2010					
**Village**	**Deaths**	**Person Years**	**Standardised Mortality Rate / 1000 PY [1]**	**90 % confidence Interval**	**Deaths**	**Person Years**	**Standardised Mortality Rate / 1000 PY [1]**	**90 % confidence Interval**	**Rate Ratio 09-10/07-08**	**90 % confidence interval**
**A**	74	10169	7.24	5.85 - 8.62	48	9814	4.75	3.62 - 5.88	0.66	0.48 - 0.89
**B**	34	4634	7.41	5.32 - 9.51	31	4495	7.14	5.03 - 9.25	0.96	0.64 - 1.44
**C**	45	7717	6.12	4.61 - 7.63	45	7484	6.13	4.60 - 7.65	1.00	0.71 - 1.41
**D**	30	5126	5.90	4.13 - 7.68	19	4945	3.84	2.39 - 5.30	0.65	0.40 - 1.06
**E**	22	3886	5.93	3.84 - 8.01	12	3806	3.19	1.67 - 4.72	0.54	0.30 - 0.97
**F**	40	5255	7.53	5.56 - 9.49	28	4963	6.44	4.39 - 8.50	0.86	0.58 - 1.27
**G**	21	3423	6.22	3.99 - 8.45	13	3263	4.14	2.25 - 6.04	0.67	0.38 - 1.18
**H**	62	7023	9.21	7.27 - 11.14	24	6786	3.49	2.32 - 4.66	0.38	0.26 - 0.56
**I**	42	6704	6.37	4.75 - 7.99	27	6313	4.48	3.06 - 5.90	0.70	0.47 - 1.05
**J**	40	6333	6.39	4.72 - 8.06	30	5923	5.46	3.77 - 7.14	0.85	0.58 - 1.26
**K**	37	9670	3.90	2.84 - 4.96	40	9513	4.21	3.11 - 5.31	1.08	0.74 - 1.57
**L**	23	3058	7.87	5.16 - 10.58	10	3045	3.40	1.63 - 5.17	0.43	0.23 - 0.80
**M**	41	4707	8.94	6.64 - 11.24	26	4477	5.69	3.85 - 7.53	0.64	0.42 - 0.96
**N**	20	2596	7.97	4.96 - 10.99	14	2506	5.31	2.98 - 7.63	0.67	0.37 - 1.19
**O**	39	4165	9.59	7.05 - 12.13	24	4021	6.33	4.18 - 8.49	0.66	0.43 - 1.00
**P**	37	5882	6.34	4.63 - 8.06	24	5724	4.52	3.00 - 6.04	0.71	0.47 - 1.09
**Q**	16	2633	5.97	3.52 - 8.43	21	2619	8.07	5.16 - 10.97	1.35	0.78 - 2.34
**R**	10	1490	7.15	3.33 - 10.98	9	1421	6.60	2.93 - 10.27	0.92	0.44 - 1.93
**S**	11	1414	7.93	4.00 - 11.85	10	1462	6.82	3.25 - 10.39	0.86	0.42 - 1.76
**T**	9	1329	6.90	3.14 - 10.66	7	1324	5.57	2.10 - 9.04	0.81	0.36 - 1.83
**U**	14	2133	6.75	3.63 - 9.88	8	2210	4.74	1.58 - 7.91	0.70	0.36 - 1.37
**V**	29	4459	7.01	4.85 - 9.17	47	7932	6.09	4.62 - 7.56	0.87	0.59 - 1.27
**W**	12	1724	8.03	4.07 - 11.98	11	3088	3.51	1.77 - 5.24	0.44	0.22 - 0.85
**X**	14	1627	8.63	4.85 - 12.42	18	2708	6.76	4.13 - 9.40	0.78	0.44 - 1.41
**Y**	6	823	10.39	3.10 - 17.67	6	1464	3.88	1.21 - 6.55	0.37	0.15 - 0.91
**Overall**	**728**	**107980**	**6.88**	**6.48 - 7.32**	**552**	**111306**	**5.12**	**4.75 - 5.48**	**0.70**	**0.64 - 0.77**

[1] PY = Person years of observation

Table S2 Analysis of Non AIDS/TB mortality rates and rate ratios aggregated by village for 2007-2008 and 2009-2010

	2007 – 2008				2009 – 2010					
**Village**	**Deaths**	**Person Years**	**Standardised Mortality Rate / 1000 PY [1]**	**90 % confidence Interval**	**Deaths**	**Person Years**	**Standardised Mortality Rate / 1000 PY [1]**	**90 % confidence Interval**	**Rate Ratio 09-10/07-08**	**90 % confidence interval**
**A**	68	10169	6.43	5.14 - 7.72	59	9814	5.72	4.49 - 6.96	0.89	0.66 - 1.20
**B**	22	4634	4.75	3.07 - 6.43	39	4495	8.74	6.44 - 11.05	1.84	1.19 - 2.86
**C**	51	7717	6.70	5.14 - 8.26	48	7484	6.50	4.92 - 8.07	0.97	0.70 - 1.35
**D**	30	5126	5.74	4.02 - 7.47	32	4945	6.51	4.61 - 8.41	1.13	0.74 - 1.73
**E**	25	3886	6.62	4.44 - 8.80	22	3806	6.00	3.89 - 8.10	0.91	0.56 - 1.46
**F**	29	5255	6.06	4.18 - 7.94	26	4963	5.75	3.84 - 7.66	0.95	0.62 - 1.45
**G**	22	3423	6.72	4.38 - 9.06	29	3263	8.94	6.23 - 11.65	1.33	0.84 - 2.11
**H**	43	7023	6.52	4.85 - 8.19	54	6786	8.61	6.63 - 10.59	1.32	0.95 - 1.83
**I**	37	6704	5.53	4.03 - 7.03	38	6313	5.46	3.99 - 6.93	0.99	0.67 - 1.46
**J**	42	6333	7.50	5.55 - 9.45	29	5923	5.72	3.92 - 7.53	0.76	0.53 - 1.11
**K**	48	9670	5.19	3.96 - 6.43	43	9513	4.60	3.43 - 5.77	0.89	0.63 - 1.25
**L**	14	3058	4.25	2.34 - 6.15	18	3045	5.82	3.52 - 8.12	1.37	0.75 - 2.50
**M**	26	4707	5.00	3.36 - 6.64	22	4477	5.27	3.40 - 7.14	1.05	0.65 - 1.70
**N**	28	2596	13.22	9.00 - 17.45	14	2506	5.54	3.13 - 7.95	0.42	0.25 - 0.71
**O**	22	4165	5.48	3.53 - 7.43	30	4021	8.06	5.58 - 10.55	1.47	0.94 - 2.31
**P**	41	5882	7.12	5.29 - 8.95	37	5724	6.05	4.41 - 7.69	0.85	0.58 - 1.24
**Q**	11	2633	4.02	1.99 - 6.05	11	2619	3.86	1.90 - 5.81	0.96	0.46 - 1.98
**R**	9	1490	6.40	2.82 - 9.99	9	1421	6.15	2.70 - 9.60	0.96	0.44 - 2.08
**S**	6	1414	4.32	1.40 - 7.25	6	1462	3.86	1.24 - 6.49	0.89	0.34 - 2.34
**T**	7	1329	5.72	2.19 - 9.26	5	1324	4.25	1.16 - 7.33	0.74	0.30 - 1.86
**U**	10	2133	5.65	2.22 - 9.07	12	2210	9.41	4.10 - 14.71	1.67	0.92 - 3.03
**V**	19	4459	4.46	2.75 - 6.17	40	7932	5.11	3.76 - 6.45	1.14	0.73 - 1.80
**W**	7	1724	3.70	1.41 - 5.99	19	3088	6.38	3.88 - 8.88	1.73	0.81 - 3.66
**X**	13	1627	9.00	4.91 - 13.08	14	2708	4.92	2.74 - 7.09	0.55	0.29 - 1.02
**Y**	5	823	6.31	1.66 - 10.96	13	1464	8.12	4.34 - 11.89	1.29	0.54 - 3.06
**Overall**	**635**	**107980**	**6.06**	**5.66 – 6.45**	**669**	**111306**	**6.11**	**5.71 - 6.45**	**1.01**	**0.92 – 1.10**

[1] PY = Person years of observation

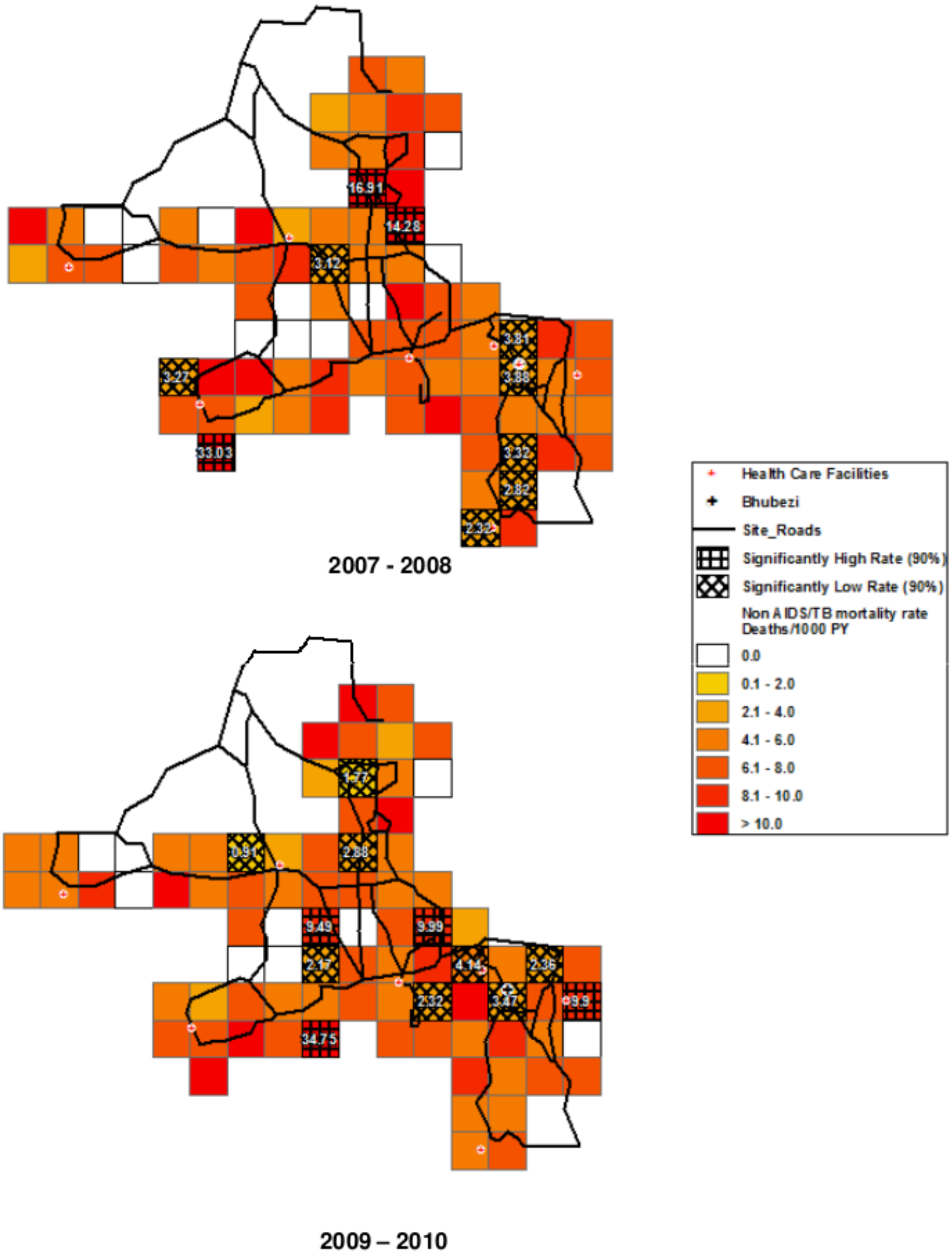

Supplementary Figure 1 Non AIDS/TB mortality rates (deaths per 1000 PY) from 2007 to 2008 (upper panel) and 2009 – 2010 (lower panel) in 2km grid squares for the Agincourt HDSS site [1,2]

[1] For grid squares with significantly low rates, the upper bound of the 90 % confidence interval is less than the mean rate. The label is the mortality rate for the grid square

[2] For grid squares with significantly high rates the lower bound of the 90 % confidence interval is greater than the mean rate. The label is the mortality rate for the grid square

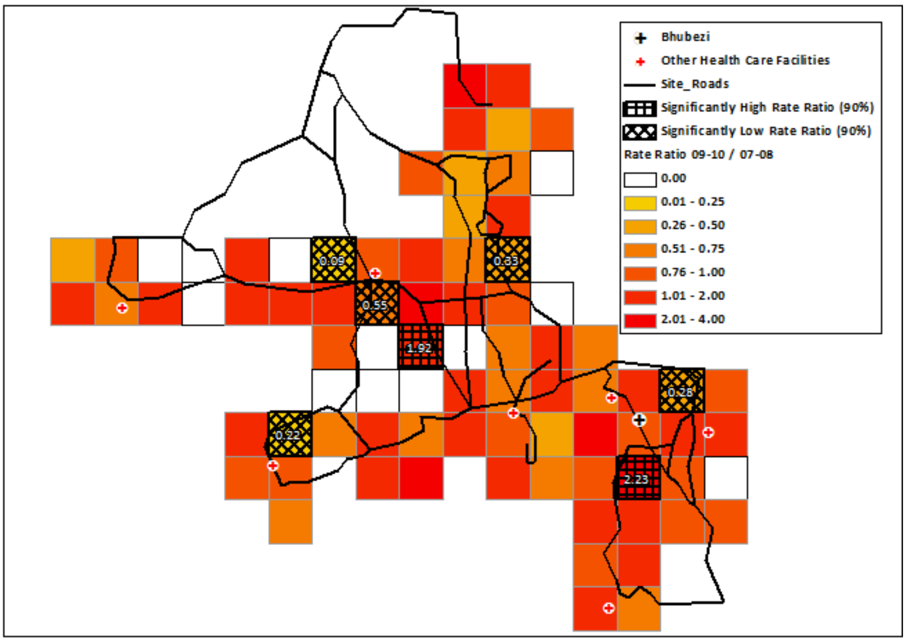

Supplementary Figure 2 Non AIDS/TB mortality rate ratios for 2009 – 2010 compared to 2007 – 2008 for the Agincourt HDSS site [1,2]

[1] For grid squares with significantly low rate ratios, the upper bound of the 90 % confidence interval is less than the mean rate ratio

[2] For grid squares with significantly high ratios, the lower bound of the 90 % confidence interval is greater than the mean rate ratio
